# Supplementary material for: Are carbon emissions trading and green financial instruments synergistic? -Comprehensive quantitative research based on content analysis
Source: PLoS One. 2024 Mar 7;19(3):e0298601. doi: 10.1371/journal.pone.0298601 (PMC10919601; doi:10.1371/journal.pone.0298601)
Supplement: S1 Data — (ZIP) [file pone.0298601.s001.zip › Data/地级市Z_F工作报告.zip!/╔╜╢1⁄2/╘μ╫»╩╨/└┤╫╘╣1⁄2╓┌║┼í╛┬φ┐╦╩2╛▌═°í┐_2017.docx]

各位代表：

现在，我代表市人民政府向大会报告工作，请予审议，并请市政协各位委员提出意见。

一、过去五年工作回顾侯卫东官场笔记

2012年以来，面对错综复杂的宏观经济形势和持续加大的下行压力，在市委的正确领导下，全市人民紧紧扭住发展不放松，凝心聚力，攻坚克难，坚定不移加快城市转型，取得了经济社会发展的新成就。综合实力持续提升。2016年，全市生产总值2142.6亿元，公共财政预算收入147.4亿元，社会消费品零售总额892.3亿元，分别是2011年的1.4倍、1.5倍和1.9倍。城镇、农村居民人均可支配收入达到27708元和13018元，年均分别增长8.4%、10.5%。五年间，固定资产投资累计完成7127亿元，是上一个五年的2.3倍。产业结构持续优化。三次产业比例由8.1∶58.9∶33调整为7.6∶51.2∶41.2。整建制认定为国家现代农业示范区。煤化工产业实现了从初级产品到煤基新材料的迈进，新兴产业加速膨胀，高新技术产业产值、非煤产业增加值占比分别提高8.2个、15.9个百分点。限上服务业企业发展到1329家，旅游综合收入年均增长13.7%。改革开放持续深化。进一步简政放权，共取消和调整市级行政权力事项1679项；市场主体发展到30万家，是2011年的1.7倍。市属工业企业改革改制基本完成。承包地“三权分置”、新型农村合作金融等改革经验在全国、全省推广。五年间，共引进世界和全国500强企业项目55个，完成进出口67.8亿美元。开发区体制机制改革迈出实质性步伐。枣庄高新区晋升为国家级高新区。城乡面貌持续改观。枣庄市城市总体规划获得国务院批复，“一主一副两区多点”的城镇格局正在形成。户籍人口、常住人口城镇化率分别达到44.6%、55.5%。城市基础设施投资累计完成172亿元，整治背街小巷300余条，新建改造各类地下管网1148公里，供热面积是五年前的2.7倍。全市公路通车里程达到8440公里，BRT实现五区通达、无缝换乘。顺利完成13个省市级新型城镇化示范镇建设任务，722个村达到美丽乡村创建标准，城乡环卫一体化实现市域全覆盖。代表山东省参加迎淮核查，取得淮河流域和全国九大流域考核“双第一”。创建成为国家森林城市、国家园林城市。民生福祉持续增进。五年间，完成民生支出781.5亿元，占公共财政总支出的71.3%。累计新增城镇就业26.5万人，农村劳动力转移就业31.7万人。城乡居民基础养老、企业退休人员养老、城市和农村低保标准分别是2011年的1.8倍、1.6倍、1.7倍和2.3倍。累计开工建设保障性安居工程6.9万套。新建改建中小学标准化校舍190万平方米。北京中医药大学枣庄医院建成运营，全市医疗机构床位数增加40%。成功举办首届中国年博会、第二届中国非博会、第三届全国智运会。平安枣庄、食安枣庄建设扎实推进。政府自身建设持续加强。深入开展党的群众路线教育实践活动、“三严三实”专题教育和“两学一做”学习教育，政风行风进一步好转。自觉接受人大、政协监督，人大代表建议和政协提案全部按时办结。建立健全政府议事规则、决策咨询、法律顾问、新闻发布、政务公开等制度，政府工作的民主化、法治化、科学化水平不断提高。

各位代表，刚刚过去的2016年，全球经济在困境中低迷徘徊，国内经济在阵痛中换挡调速，枣庄转型发展的压力前所未有。我们秉持“干在当下、成在实处”的理念，顽强拼搏，负重奋起，经受住了经济寒冬的考验，实现了“十三五”的稳健开局。

（一）抓紧抓实了实体经济。深入实施“265”产业集群培育工程，中材科技锂膜材料、东方光源二期等一批产业项目开工建设或投产运营。全力培植大数据产业，成为全省第三个互联网一级节点城市，鲁南数据中心主体工程基本建成，互联网小镇入驻运营企业43家，我市被认定为“宽带中国”示范城市。工业技改完成投资439亿元，增长17.6%，增幅居全省首位。过剩产能化解率80%以上，煤炭、水泥、轮胎、玻璃产能利用率稳步提高。继续实施领导干部联系帮包产业企业制度，推行大用户直供电等一系列降成本举措，共为企业减负15亿元。高效使用接续还贷资金，全年累计拨付7.2亿元。服务业增加值占比提高1.5个百分点。农村土地规模经营比例达到53％，家庭农场、市级以上农业龙头企业分别发展到983家和352家。实体经济提质增效，在转型振兴道路上又前进了一步。

（二）引进实施了一批重点项目。精准开展招商引资，成功举办北京、长三角等招商周活动，新开工外来固定资产投资亿元以上项目139个，其中10亿元以上项目11个。57个市级重点项目完成投资236亿元，十电扩建第一台机组、八一热电二期一号机组、瑞达聚丁烯一期、万达广场等18个项目基本建成。筹划多年、总投资30多亿元的庄里水库，坝基灌浆即将完工，移民安置顺利推进，已完成投资17.6亿元；总投资约70亿元的抽水蓄能电站项目正在争取纳入国家选点规划。期盼已久的枣庄机场，经国务院同意，纳入全国民用机场布局规划。峄州港建成运营，万年闸复线船闸、沂台线南延、新城至滕州BRT等项目加快推进，枣木高速东延、枣菏高速等工程开工建设。建成海绵城市6.4平方公里，开工地下综合管廊12.2公里。随着一批大项目、好项目的实施，枣庄长远发展的基础更加稳固。

（三）有效激活了创新动能。产学研合作实现重大突破，与浙江大学合作建设山东工业研究院，与北京航空航天大学合作设立机床创新研究院，与北京理工大学合作成立鲁南工业技术研究院，与清华大学合作建设孵化器2.0科技产业园，厚植了发展优势。建成科技企业孵化器和众创空间15个，市级以上创业示范园区9个。目前，全市共引进“千人计划”“万人计划”“泰山学者”等高层次人才25人，确定首批“枣庄英才”24人。新增专利授权2531件、山东名牌产品10个、山东服务名牌3个。大力推进企业股改上市，24家企业完成股份制改造，丰元股份成功登陆深交所，我市已有2家企业在A股上市、6家企业在“新三板”挂牌。威海商业银行、亚太财险、国泰君安证券等6家金融机构进驻枣庄。创新驱动发展的共识进一步凝聚，经济增长动能加速转换。

（四）改善提升了生态环境。深入开展大气污染综合治理百日攻坚行动，依法关停整治露天储煤场66家、砖瓦企业87家、港口码头52家；变季节性秸秆禁烧为全年禁烧，是全省4个无卫星火点城市之一；“蓝天白云”天数达到207天，获得省级补偿资金2134万元、居全省第3位。提前完成消除劣五类水体省定任务，基本实现城市建成区污水管网全覆盖；加强周村、羊庄等水源地保护，全面关闭拆除饮用水源保护区内畜禽养殖场。建立区域山体名录，严控开山采石。新增造林12.9万亩，新建提升绿色生态廊道356公里，山亭、台儿庄纳入国家重点生态功能区。10个村入选国家和省传统村落名录，5个村入选国家和省美丽宜居村庄，2个镇被评为省宜居小镇。自然生态正在成为最靓丽的城市名片，枣庄变得更加宜居宜业。

（五）扎实办好了民生实事。各级财政投入2.4亿元，全力推进精准扶贫，5.6万人实现脱贫。开展创业就业培训3万余人次，直接扶持创业2677人。综合施策化解城镇中小学“大班额”，累计完成投资38.4亿元，新建学校18所、改扩建105所。全面推开公立医院综合改革，实行人员总量控制备案管理，参与改革的22家医院全部取消药品加成。市妇保院新院建成启用。深入实施“气化枣庄”工程，全市所有镇街通上了天然气。改造农村危房1545户。完成农村改厕13万户，新建改建旅游厕所919座，提前完成全省唯一全域旅游厕所革命示范市建设任务。新建改造农村公路437公里，683个村居实现“户户通”。以创建国家卫生城市为抓手，全面清理城区“十乱”，改造老旧小区109个、农贸市场13处，拆除违章建筑4500多处，得到了市民的普遍支持认可。供销社综合改革代表山东省通过国家评估验收。民族宗教、外事侨务、妇女儿童、红十字、老龄、残联、史志、人防、地震、气象等事业也都取得了新进展。

各位代表！成绩的取得，得益于省委、省政府和市委的正确领导，离不开人大、政协和社会各界的监督支持，归功于全市人民的众志成城、发奋图强。在此，我代表市政府，向奋斗在各行各业的广大党员干部群众，向解放军、武警驻枣官兵和中央、省驻枣单位，向在我市创业发展的投资者、建设者，向所有关心、支持枣庄发展的港澳台同胞和海内外朋友，表示衷心的感谢和崇高的敬意！

同时，我们也清醒地认识到，我市发展中还存在不少困难和问题。受经济下行影响，一些企业经营困难，民间投资、制造业投资增长缓慢，外贸出口增幅回落，工业经济、现代服务业发展不快。高端人才、创新平台稀缺，新上大项目、好项目较少，新兴税源、骨干税源缺乏，县域经济实力不强。城乡基础设施还有诸多欠账，小城镇建设缺少特色，大新城辐射带动能力不足。安全生产形势依然严峻，雾霾治理和水质改善任重道远，食品药品安全保障水平有待提高，教育、医疗、文化等基本公共服务不均衡。部分公务人员缺乏担当精神、服务观念和规矩意识，全面从严治党、从严治政仍需不断加强。对此，我们将坚持问题导向，务实研究解决，补齐发展短板，让广大群众年年都能看到新变化、得到新实惠。

二、今后五年政府工作目标和2017年重点任务

今后五年是全面建成小康社会，实现第一个百年梦想的决胜阶段。当前，世界经济在深度调整中曲折复苏，新一轮科技和产业革命蓄势待发。纵观国内，经济发展进入新常态，新发展理念正在引领经济步入新阶段。今年，将召开党的十九大和省第十一次党代会，谋划全国、全省未来五年的发展蓝图。刚刚闭幕的市第十一次党代会，明确了枣庄今后五年的发展目标和路径，描绘了城市转型的新愿景新希望。好风凭借力，扬帆正当时。让我们以坚如磐石的忠诚之心，以饮水思源的感恩之情，以事不避难的担当之责，背起行囊、挺起脊梁，撸起袖子、甩开膀子，奋力开创更加幸福美好的未来。

今后五年政府工作的指导思想是：高举中国特色社会主义伟大旗帜，以邓小平理论、“三个代表”重要思想、科学发展观为指导，深入学习贯彻习近平总书记系列重要讲话精神和治国理政新理念新思想新战略，坚决维护以习近平同志为核心的党中央权威，统筹推进“五位一体”总体布局和协调推进“四个全面”战略布局，自觉践行新发展理念，按照省委“一个定位、三个提升”要求，紧紧围绕“在全面建成小康社会进程中走在前列”，牢牢把握转型升级和经济文化融合发展“两大高地”战略定位，着力推动创新、开放、生态“三个跨越”，加快构建现代产业、新型城镇、社会治理和民生保障“四大体系”，干在当下、成在实处，努力打造自然生态宜居宜业新枣庄。今后五年，我们的主要奋斗目标是：生产总值年均增长7.5%左右，公共财政预算收入年均增长6.5%左右，城乡居民人均可支配收入年均增长8%左右，提前实现经济总量和居民人均收入比2010年翻一番，与全省同步全面建成小康社会。

2017年是实施“十三五”规划的重要一年和推进供给侧结构性改革的深化之年。我们要坚持稳中求进工作总基调，以提高发展质量和效益为中心，以推进供给侧结构性改革为主线，牢固树立和贯彻落实新发展理念，适应引领经济发展新常态，紧扣转型升级、改革创新两大主题，突出项目建设、安全发展两大支撑，优先发展工业经济、县域经济两大领域，不遗余力守住生态环境、民生福祉两大底线，推动全市经济社会始终在健康可持续的轨道上运行。建议今年全市经济社会发展的主要预期目标为：生产总值增长7%左右；公共财政预算收入增长4%左右；固定资产投资增长10.5%左右；社会消费品零售总额增长10.5%左右；进出口保持稳定；城镇居民人均可支配收入增长7.5%左右，农村居民人均可支配收入增长8.5%左右；居民消费价格上涨3%左右；全面完成省下达的节能减排降碳约束性指标。工作中，突出抓好以下七个方面：

（一）致力产业转型升级，加快振兴实体经济。坚持从供给侧和需求侧两端发力，推进传统产业做优、新兴产业做大、三次产业融合，全方位聚焦实体经济发展。

一是突出“四大板块”，着力推进工业创新发展。围绕工业强市，紧密对接中国制造2025，启动实施12个重点产业五年发展规划，培育壮大“265”产业集群。高端装备制造板块。依托万户数控、威达重工等企业，实施航空发动机叶片阵列机床研发、工业机器人关键部件生产线等项目，提升高端数控机床研发制造水平。加快东方光源光纤预制棒、中材科技锂膜材料、维信诺OLED等项目建设，拓展高端装备市场。支持腾拖大马力拖拉机、亿和精密铸造、普鲁特车铣加工等项目建设，补强机械装备、基础部件等产业链薄弱环节。化工新材料和精细化工板块。深入开展化工产业转型升级专项行动，加快化工园区重新审核认定，推动企业入园发展、安全发展。依托联泓新材料、潍焦薛城能源、盛隆能源等龙头企业，重点推动甲醇制烯烃技改、20万吨轻质油、焦炉煤气发电等项目建设，进一步优化产业和产品结构。加快泰和年产28万吨新型水处理剂项目建设，做强环保药剂行业领军企业。推动丰元股份老厂区搬迁改造，抓好精制草酸、磷酸铁锂等项目，打造以上市促转型的标杆。医药和医疗器械板块。支持苏鲁海王医药集团发展壮大，深化与康力医疗器械的合作，加快推进医疗耗材和康复保健器械产业基地建设；依托三九、益康等企业加强新药物研发，支持威智手性制剂、杰诺新型酶制剂等项目建设，扩大市场份额，提升行业竞争力。大数据产业板块。建成运营鲁南数据中心一期工程，推进泰盈、阳光保险呼叫中心建设，发挥互联网一级节点优势，全力突破大数据产业。坚持高点定位，抓好互联网小镇建设运营，打造富有创新活力的高铁信息产业园。围绕“智慧枣庄”建设，搭建政务综合数据、无线网络覆盖等平台，打造教育、医疗、旅游、交通等16朵“智慧云”，促进城市治理更加高效。坚持把技改作为工业经济内涵式发展的有效途径，启动新一轮高水平技术改造，依托新技术、新业态，全面提升煤电、轮胎、纺织、造纸、食品等传统产业。抓好十电扩建第二台机组、田陈煤矸石综合利用、今缘春酒业扩建、恒宇特种纸等重点技改项目，力争工业技改投资增长12%以上。

二是突出“四个一批”，着力推进现代服务业发展。坚持提升传统服务业和培育新兴服务业并举，推动生产性服务业向专业化拓展、生活性服务业向高品质迈进。实施一批全域旅游项目。大力推进滕州、山亭、台儿庄创建国家全域旅游示范区，争创全域旅游示范市。抓好台儿庄古城、冠世榴园等重点景区管理提升，推动微山湖红荷湿地、抱犊崮-熊耳山创建5A级景区，实施祥和庄园、石佛山寨等文化旅游项目，策划推出运河游、红色游等10条精品线路，发展乡村民宿、休闲体验等旅游新业态，努力构建“山水呼应、人文交融、全域覆盖、悠游枣城”的旅游发展格局。打造一批城市特色商圈。抓好万达广场、中央广场等项目建设运营，打造商业新地标。推进接官巷、和顺老街等商业街区建设，形成特色鲜明、业态集聚的商贸功能区。培育壮大真爱商城、金源建材装饰大世界等专业市场，加快实体零售创新转型，拓展仓储配送、展会信息、网络营销等服务功能，增强区域辐射力和吸引力。壮大一批现代物流园区。依托运河黄金水道，着力推进台儿庄港航物流园、峄州港航产业园、枣庄铁路物流园等项目建设，推动沿运经济带加速隆起。加快市粮食仓储物流中心、通汇农副产品物流中心、张范汽车物流产业园等项目建设，推进物流产业特色化、专业化、集聚化。培育一批新兴服务业态。抓好鲁南电商谷、枣庄电商产业园、滕州阿里巴巴农村淘宝服务中心等项目，鼓励发展环保、科技服务业，搭建智能家政服务平台，培育更多新的消费热点。

三是突出“五化联动”，着力推进现代农业发展。以国家农村改革试验区、现代农业示范区建设和国家农业可持续发展试验示范区创建为引领，加快农业供给侧结构性改革，推动一产“接二连三”，大力发展“新六产”，提高农业供给质量。推进农业产业化。重点发展以家庭农场为主的适度规模经营，扶持马铃薯、石榴、大枣等农业示范基地10处，新认定现代农业示范园30家。发展农产品精深加工，积极开发方便食品、休闲食品、马铃薯主食产品，扶持壮大一批农业龙头企业、加工型专业合作社。推进农业标准化。加快畜禽养殖场标准化改造，新认定一批市级以上标准化生产基地。健全农产品质量安全追溯体系，完善13处智能监测点，构建全覆盖的农产品监管信息网络。落实“菜篮子”市长负责制，保障菜品市场供应安全。推进农业品牌化。实施农业品牌建设年活动，鼓励企业开展品牌创建，支持春藤黑盖猪等申报国家地理标志产品，新认证“三品一标”40个。整合提升传统名优农产品资源，打造一批具有枣庄特色的公用品牌。推进农业高效化。坚持藏粮于地、藏粮于技，加强高标准农田建设，开展绿色增产和粮食高产创建、新品种展示示范、良种良法配套、先进农机具技术推广与服务。抓好农业物联网示范工程，在100个村开展农业信息进村入户试点。办好枣庄新型职业农民学院，深入实施职业农民培育“百千万”工程。推进农业可持续化。落实最严格的耕地保护制度，守住耕地红线。发展生态农业、循环农业、节水农业，开展化肥农药“零增长”行动，实施水肥一体化、土壤改良修复、病虫害生物防治、秸秆还田利用等工程，增强农业综合生产能力。

（二）致力招商引资上项目，加快壮大县域经济。启动实施县域经济发展三年提升行动计划，优化产业布局，强化要素保障，提高双向开放水平，筑牢“两大高地”建设的基石。

一是抓招商重落地。研究制定适应经济新形势的招商引资政策，围绕培育“265”产业集群，策划包装一批重大产业项目和基础设施项目，瞄准先进地区、发达国家和大企业集团，办好北京、长三角、香港等招商周活动，精准开展产业招商。突出企业主体作用，借助浙江、福建等在枣商会的商脉资源，务实开展以商招商。继续实行招商引资市级领导帮包责任制，完善重大项目落地推进机制，建立涵盖项目成长全过程的政策支持体系，确保早落地、早建成、早达效。力争新开工过5亿元项目20个以上，过20亿元项目5个以上。认真对接上级产业、财税、金融等政策，加大项目和资金争取力度，助推枣庄加快转型发展。

二是抓园区育集群。加快省级开发区体制机制改革创新，赋予独立的人财事管理权限，探索职员制、岗位绩效工资制等市场化运行模式。建立做大投融资平台，完善供气、供热、污水处理等配套设施，提升项目承载能力。对全市开发区统一进行“二次规划”，明确产业定位，促进园区差异化、特色化发展。在市级层面建立项目统筹、利益分享、反向约束机制，推进产业集聚发展，把园区打造成为县域经济的“发动机”。开展节约集约用地评价，探索存量建设用地“二次开发”机制，清理处置“空壳”企业、“僵尸”企业，引导镇街项目和企业进区入园。支持枣庄高新区创建国家高新技术产业标准化示范区，强力推动滕州经济开发区创建国家级园区。

三是抓平台促外贸。抢抓“一带一路”战略机遇，积极融入国家对外开放大格局。大力开展“商务平台建设年”活动，依托山东兄弟、秦工国际等企业，尽快做大外贸综合服务平台，帮助更多中小微企业开展对外贸易，培植新的外贸增长点；建立外贸公共信息平台，拓展阿里巴巴一达通业务，引导传统外贸企业和电商企业发展跨境电子商务，让广大企业不出家门就能畅游全球市场。继续开展零出口企业“唤醒行动”，加大骨干出口企业扶持力度，推动辛化硅胶、艾菲尔管件、万宝童车等小品牌闯出大天地。鼓励先进技术、关键设备进口，支持企业积极稳妥“走出去”开发资源、投资建厂、承揽工程，带动产能合作。

四是抓企业强支撑。启动“个转企提升年”活动，力争2500家个体工商户转为企业。引导中小企业、民营企业特色发展，实施“一企一技术”提升工程，培育“单项冠军”企业4家、“专精特新”企业30家、创新成长型企业40家。以更大力度推动企业股份制改造，完成30家股改任务。鼓励企业在“新三板”和区域性股权交易中心挂牌，到主板、创业板和境外资本市场上市，争取泰和水处理A股上市、耀国光热境外上市；力争通过几年的努力，实现上市企业县域全覆盖。坚持质量强企、品牌兴企，引导企业发扬工匠精神，融入国家品牌计划、中国品牌影响力提升计划和“好品山东”平台，新申请马德里国际商标5件以上。

（三）致力重点领域改革，加快释放发展活力。发挥改革的牵引作用，以重大改革的“一子落”，带动经济棋局的“全盘活”。

一是深入落实供给侧结构性改革重点任务。“去产能”方面，推动产能过剩、工艺落后和耗能高、污染重的企业主动退出、优化整合、改造提升，完成省定过剩产能化解任务。压减煤炭产能30万吨。“去库存”方面，坚持控增量、提质量、去存量，让市场稳得住、百姓有房住。引导非城市居民进城购房，支持开发商发展复合型地产，鼓励土地功能混合，消化商业营业用房库存18万平方米，促进房地产行业转型升级。引导企业压缩“两项资金”占用，创新工业品产销模式，运用大数据挖掘市场需求，产销率保持在99%左右，实现供给与消费高效互动。“去杠杆”方面，通过债转股、投贷联动、股权融资、资产证券化等方式，着力降低企业杠杆率。完善金融监管机制，加大资金链、担保圈等风险防控，畅通资金流向实体经济的渠道。规范政府举债方式和行为，优化区域金融环境。“降成本”方面，切实提高接续还贷资金使用效益，清理违规涉企收费，降低社保综合费用，继续推行大用户直供电，落实小微企业和高新技术企业优惠政策，减轻税费负担和生产成本。“补短板”方面，围绕提升创新能力、引进高端人才、完善基础设施、优化公共服务、营造良好环境等领域，把薄弱环节补强，让发展更有底气、更有后劲。

二是深化“放管服”改革。继续简政放权，凡国家和省取消的审批事项，以及外地不审批的同类事项，我市一律不再审批；清理规范行政审批前置事项，再削减一批中介服务收费项目，深化建设项目区域化评估评审试点，探索推进投资建设领域多图联审、多评合一。继续强化事中事后监管，实现“双随机、一公开”全覆盖；加快社会信用体系建设，对严重违法失信主体实行市场禁入，形成“一处失信、处处受限”的惩戒机制；完成行业协会商会与行政机关脱钩任务。继续优化服务，编制公开公共服务事项目录和服务指南，清理规范证照年检和指定培训，精简各类证明、盖章和申请材料，让企业和群众办事更便捷。

三是不断完善投融资体系。抓好财金控股、基础设施投资、鲁南（枣庄）经济开发投资等平台管理运营，增强滚动发展和资金供给能力；整合城市基础设施、场馆设施、农林水港等资源，搭建若干国有资产融资平台，总规模不低于500亿元，激活沉睡的国有资产，置换涌动的市场资本。做强市股权投资引导基金，围绕智能制造、医疗健康、文体旅游等设立子基金，撬动更多民间投资支持产业发展；设立政府和社会资本合作发展基金，加大推广力度，促进成熟项目尽快落地。加快推进国开行、农发行、邮储银行等签约项目信贷投放，保障重点项目建设需求。大力引进股份制银行、保险公司等金融机构，壮大枣庄银行、枣庄农商银行实力。各区分别成立1家注册资本金3亿元以上的融资担保机构，滕州成立1家注册资本金5亿元以上的融资担保机构。

四是稳步推进国企国资改革。积极发展混合所有制经济，优先以增量方式吸引各类资本，参与国有企业改制重组和共同投资。以泉兴集团、城市供水等市属企业为主体，探索建立国有资本投资运营公司或产业投资公司。完善现代企业制度，市属国有企业全面建立公司法人治理结构，开展董事会选聘职业经理人试点，逐步建立国有企业专职外部董事、外部监事和财务总监委派制度。推进市属经营性国有资产集中统一监管，加快构建以管资本为主的国有资产管理体制。坚持管资本与管党建相统一，切实发挥党组织在国有企业改革发展中的主导作用。

五是加快农村改革步伐。深化农村产权制度改革，加强农村土地承包经营权流转监督管理，加快农民房屋权属登记，全面完成农村集体建设用地和宅基地使用权确权；鼓励开展资源变资产、资金变股金、农民变股东改革，农村集体产权制度改革试点村达到300个以上，促进更多农村产权进入市场交易，力争农村改革继续走在全国前列。全面完成国有林场改革任务，形成可持续发展的长效机制。扩大社区金融互助协会、村级融资担保基金试点，两年内新型农村合作金融试点覆盖所有镇街。推进政府购买农业公益性服务试点，拓展农产品目标价格保险范围，织牢农业生产“保护网”。

（四）致力科技人才建设，加快形成创新创业支撑体系。深入实施创新驱动发展战略，大力推进以科技和人才为核心的全面创新，做实创新创业载体，全力培育发展新动能。

一是在推动产学研合作方面下功夫。加强与中科院的战略合作，加快建设化工新材料技术创新与产业化基地，带动我市化工产业层次整体跃升。抓好浙江大学山东工业研究院、北京理工大学鲁南工业技术研究院、北京航空航天大学机床创新研究院建设，引导更多园区和企业联姻知名高校、科研院所，设立产学研联合体或战略联盟，通过技术转移嫁接，让企业“开大花、结硕果”。支持企业建设重点实验室、工程实验室等研发机构，新建省级示范工程技术研究中心1家、省级企业技术中心4家。围绕锂电、机床、煤化工、玻璃、纺织等领域，重点布局提升一批检验检测平台和中试基地，争创一批产业协同创新中心、制造业创新中心，争取更多行业标准化委员会落户枣庄。用好专利专项资金，放大科技创新券、产业基金引导功能，激发创新创造活力。

二是在推动人才集聚方面下功夫。深入实施“枣庄英才”集聚工程，重点引进急需紧缺专业人才，以及拥有核心技术、研创能力的高层次人才和团队。引进产业领军人才、金融高管、特聘专家等高层次人才50名，评选表彰“枣庄英才”20名。打破编制身份限制，实行更加柔性开放的人才政策，广开门庭迎人才，海纳百川聚人才。深化职称制度改革，建立以能力、业绩和贡献为导向的人才评价体系，切实用好本土人才。依托职业院校、领军企业，实施高技能人才提升工程，开展现代学徒制试点，建设一支数量充足的“金蓝领”队伍。

三是在推动大众创业方面下功夫。加大创业孵化基地、众创空间、创业“梦工厂”扶持力度，打造一批省、市级创业示范园区，加快建设创业型城市。发挥创业大学的培训职能，实施大学生创业引领计划、农民工职业技能提升计划，提高创业成功率。支持返乡下乡人员到农村创新创业发展，领办创办家庭农场、农民合作社、农业产业园。探索股权激励、兼职取酬、科研成果收益分红，鼓励科技人员依靠智力创业致富。全面落实各项创业优惠政策，让更多创业者梦想成真。

四是在推动企业家队伍建设方面下功夫。把企业家群体作为重要的战略资源，大力实施企业家培育“1515”工程，用5年时间，至少培育10名国内行业领军企业家、50名省内知名企业家、100名成长型企业家、500名创新创业型青年企业家。加强企业家梯队建设，促进新老梯队顺利递接、代际之间有序传承。倡导企业家精神，开展优秀企业家评选表彰，营造企业家有作为、有地位、受尊重的良好环境。

（五）致力功能配套提升，加快城乡一体发展步伐。抓住枣庄纳入大城市试点的机遇，按照“规划整合、功能契合、产城融合、生态耦合、改革聚合”的思路，构建具有枣庄特色的新型城镇化体系。

一是突出大新城首位度。健全城市功能，提升城市品质，增强承载、集聚和带动能力。完善交通路网体系。启动祁连山路南延、长江路东延、长白山路南延等道路规划建设，抓好店韩路升级改造，构建“五横五纵一环”交通路网；加快郯兰线改造提升，推进薛微同城化发展；结合国家公交都市创建和高铁换乘枢纽建设，优化新城公交线网布局，加快公交站点建设，加强与周边地区的延伸对接，提升新城交通枢纽地位。完善教育医疗商贸体系。抓好枣庄学院新校区、市职业中专二期、市实验高中三期、龙潭实验学校、市体校新校区建设，完成市特殊教育学校迁建；加快推进市图书大厦建设，建成启用市文体中心；推动北京中医药大学枣庄医院二期、医养结合园和市医养康复中心、市妇保院二期、市立医院新院等项目，布局一批文化休闲场所、便民商业网点，汇集更多人气文气商气。完善山水生态体系。加强植树造林、护树育林，抓好巨山、龟山、凤凰山、金牛岭等山体保护，提升袁寨山省级森林公园品质；实施黑峪水库扩容、小沙河水系连通、蟠龙河提升等工程，推进高新区绿源污水处理厂项目，加快海绵城市建设，打造青山拥城、绿道环城、碧水绕城的生态空间。

二是实施重点片区棚户区改造。注重城市修补和有机更新，用好国家棚户区改造政策，重点抓好市中老火车站北、峄城前湾等53个棚改项目，开工建设棚户区2.8万套、基本建成7656套。因地制宜实施货币化安置，提高公积金使用效率，打通商品房与政策性住房转换通道。着力化解棚改遗留问题，让老百姓真正享受到棚改带来的实惠。积极推进绿色建筑和住宅产业化，创建一批国家和省级装配式建筑产业基地。

三是强化重大基础设施支撑。加快枣木高速东延、枣菏高速枣庄段、新城至滕州BRT、沂台线南延、中兴大道等工程建设，扎实推进新台高速枣庄段、世纪大道等项目。抓好万年闸复线船闸建设，实施京杭运河枣庄段二级航道整治。开展枣庄机场预可研工作，建成高铁换乘枢纽地下通道延长工程，尽快推动十电运煤专线迁建。完成峄城输变电线路改造，启动山东特高压枣庄段输变电项目。高标准推进庄里水库建设，继续做好抽水蓄能电站前期工作，开工建设马河、周村、辛庄水库增容工程，以“功成不必在我”的境界，夯实枣庄未来发展的基础。

四是因地制宜培育特色小镇。围绕产业“特而强”、功能“聚而合”、形态“精而美”、机制“新而活”，抓好西岗省级新生小城市、税郭省级重点示范镇，以及徐庄休闲慢游小镇、滨湖微山湖湿地古镇、古邵港航物流小镇等省级特色小镇创建，开展市级“十镇百村”创建活动，打造现代特色城镇新样板。启动新一轮美丽乡村创建，统筹抓好农村“七改”，改造农村危房500户，完成改厕10万户；继续实施农村公路网化工程，新建提升农村公路400公里，实现1500个村居“户户通”，把农村妆扮得更靓丽，让农民生活得更舒适。

（六）致力生态环境改善，加快打造宜居宜业家园。围绕“环境质量只能变好不能变坏”的目标，深化寻源治污、精准治污、铁腕治污，坚决向污染环境的行为“亮剑”，让优良生态成为最普惠的民生福利。

一是在聚焦治气上加力。做好“调”的文章。加快能源和产业结构调整，严控“两高”行业新增产能，坚决淘汰落后产能，推动园区循环化改造，促进工业绿色低碳发展。加速清洁能源替代利用，巩固“气化枣庄”成果，扩大镇村天然气覆盖面。抓好农村供热试点，推进集中供热向镇村延伸。做好“压”的文章。狠抓压煤减排，再削减煤炭消费56.6万吨。严格执行山东省大气污染物第三阶段排放标准限值，有序推进10万千瓦以下燃煤机组超低排放改造。深度治理民用散煤，在城中村、城乡结合部实施减煤换炉，继续开展10蒸吨以上燃煤锅炉综合治理。做好“降”的文章。抓好工业企业、城乡道路、建筑工地、餐饮油烟、机动车船等烟尘治理，加强重点行业脱硫、脱硝、除尘设施运行监管，完成30家重点工业企业烟尘整治。继续实施秸秆全年常态化禁烧。做好“管”的文章。完善重污染天气预报预警机制，落实应急管控措施，变被动“靠天驱霾”为主动“综合治霾”。夯实属地管理和行业监管责任，推行联合执法、区域执法、交叉执法，强化办案，严肃追责，让环境违法者得到严惩，让疏于管理者受到重处。

二是在巩固治水上加力。深入实施河长制，构建从水源地到水龙头的安全屏障。扎实开展涵养水源行动，完成37处农村饮用水源保护区划定。以治理总氮、总磷、氟化物等为重点，实施20个工业升级改造和污水处理设施提升项目。完善城区污水管网，用三年时间基本消除城市黑臭水体。加强重点流域、区域水污染防治，建成8处人工湿地水质净化工程，让人们重拾“蛙鸣清塘、鱼翔浅底”的美好记忆。

三是在稳步治土上加力。开展土壤环境质量调查和农用地、重点行业用地污染详查，建立污染地块名录和开发利用负面清单，完成镇街土壤国控监测点设置。继续加强农业面源污染防治，抓好危险废物处置管理、非正规垃圾堆放点整治和畜禽养殖污染治理。推行种养结合、增施有机肥等措施，促进耕地污染治理与修复，为子孙后代留下更多的沃土良田。

四是在修复生态上加力。坚持“谁开发利用、谁治理修复”和“完全开发、全面治理”的原则，强化荒山、破损山体、采矿塌陷地等生态脆弱区的保护治理，严守资源消耗上限和生态保护红线，加快省级生态市建设。深入实施国家森林城市十大提升工程，推进环城国家生态公园和绿色生态廊道建设，新增造林7万亩、绿化山体1万亩，积极创建国家生态园林城市。做好第二次污染源普查工作。推进“互联网+环保”平台建设，实现环境的精准监管与科学施治。

（七）致力办好民生实事，加快提升群众获得感。牢固树立以人民为中心的发展思想，集中财力办好惠民实事，让群众拥有满满的希望、稳稳的幸福。

一是高标准推进脱贫攻坚。继续大力实施精准扶贫、精准脱贫，确保年内实现1.55万人脱贫，省市确定的扶贫工作重点村全部退出，与全省同步基本完成脱贫攻坚任务。发挥好“第一书记”的作用，深化与威海市的扶贫协作，突出抓好产业扶贫、文化扶贫，让困难群众既富“口袋”又富“脑袋”。实施贫困村提升工程，培育壮大村集体经济，加快形成村集体持续增收、贫困户持久受益的长效机制。对没有脱贫能力的群众，政府必须义不容辞兜起来，决不让一个人在全面小康的路上掉队。

二是继续提高社会保障水平。大力实施全民参保计划，基本实现法定人员全覆盖。深化医保支付方式改革，推动居民医疗保险市级统筹，建立完善职工和居民大病保险制度，逐步提高医保待遇水平。建立健全困难群众生活保障工作协调机制，兜住基本民生底线。统筹城乡特困人员救助供养，实行保障标准与物价水平挂钩联动。稳步提高企业退休人员养老金待遇。健全农村留守儿童、老人关爱服务体系。建设15处老年人日间照料中心和30处农村幸福院，新建改造60个农村社区服务中心。实施“千企万人”就业服务计划，新增城镇就业4.2万人，农村劳动力转移就业5万人。

三是促进基本公共服务均等化。持续用力培植壮大基层财源，理顺规范市、区（市）财政事权和支出责任。按照基本民生标准、农业转移人口市民化待遇、工资性收入政策、运转经费标准和公共设施建设标准“五个基本统一”的要求，逐步构建县域基本公共服务均等化协同机制。深化基础教育综合改革，加快推进“全面改薄”，实现县域义务教育均衡发展全覆盖。全面完成城镇中小学三年建设任务，基本解决“大班额”问题。发展优质学前教育，有效破解幼教资源不足和“入托难”问题。推进健康枣庄建设，深化医药卫生体制改革，加快建立现代医院管理制度，壮大以全科医生为重点的基层卫生人才队伍。发挥北京中医药大学枣庄医院的引领作用，提高中医药传承创新能力，打造区域性中医药发展高地；拓宽合作领域，促进与健康养生产业融合发展。适应“二孩”生育高峰，深化计划生育服务管理改革。开展残疾人精准康复服务行动。继续推进国家卫生城市创建，深入治理城市违法建设，着力提高城乡环卫一体化运行效果，常态化抓好城乡环境综合整治。办好第九届市运会、第七届全民健身运动会和国际马拉松等赛事。推进市人社综合服务中心、食品药品检验中心和县级国家综合档案馆建设。建成地下综合管廊6公里。改造老旧小区108个，全面规范提升物业管理。

四是加快发展文化事业。深入实施优秀传统文化传承发展工程，根植枣庄悠久丰厚的历史文化土壤，深度挖掘墨子、鲁班、奚仲、中兴工业等地方文化，保护传承柳琴戏、运河大鼓、伏里土陶等鲁南民俗文化，整合资源叫响铁道游击队、台儿庄大战等抗战文化品牌。提升台儿庄古城国家级文化产业园、中兴文化产业园等园区的承载带动能力，创建一批“文化创意集市”，打造一批特色文艺产品，培养一批优秀文化人才，促进文化与经济深度融合。积极开展文化惠民活动，推进城乡15分钟公共文化服务圈建设，实施乡村、社区儒学推进计划，评选一批“书香之家”“书香之村”。推进“乡村记忆”工程，守护好历史文化名镇、街区、村落和非物质文化遗产。

五是加强和创新社会治理。深化平安枣庄建设，健全立体化社会治安防控体系，提升街面、社区见警率，增强群众安全感。压实信访维稳责任，完善社会矛盾纠纷常态化、多元化排查化解机制。深化“四社联动”，推行“一村一法律顾问”“一村一警务助理”制度，巩固网格化管理成果。推进安全生产领域改革发展，严格落实企业主体责任，完善安全风险分级管控和隐患排查治理双重预防体系，持续开展“大快严”集中行动，严厉打击违法开采矿产资源行为，继续实施农村公路安全生命防护工程，构筑本质安全体系。加强食品药品监管能力建设，保障群众饮食用药安全。启动第四次经济普查。抓好军地双拥共建，推进军民融合发展。

三、全面加强政府自身建设

切实增强政治意识、大局意识、核心意识、看齐意识，将全面从严治党要求贯穿从严治政的全过程，努力打造人民群众满意的服务型政府。

（一）坚持高效施政。深化“两学一做”学习教育，持续转作风、提效能、优服务。加快“政务云”建设，推动政务大数据多元集成、综合利用，让政府服务更智能。推进政务公开标准化规范化，实现决策、执行、管理、服务、结果全过程公开。加强调查研究，发挥好决策咨询智库的作用，探索建立“市民参事”制度，使政府决策上接天线、下接地气。健全新闻发布制度，提高市长热线办理质量，第一时间回应社会关切。下大气力优化营商环境，以直接服务企业和群众的部门单位为重点，开展“不作为、慢作为、乱作为”专项整治，推行电视问政、政风行风评议、社情民意调查，让“懒、庸、推、慢、浮”行为无处遁形。

（二）坚持依法行政。认真贯彻落实法治政府建设实施纲要，将政府各项工作纳入法治轨道，“十三五”末基本建成法治政府。深入开展“七五”普法和“六五”依法治市工作，发挥党政一体法律顾问制度作用，严格落实重大行政决策合法性审查、责任终身追究制度，决不让权力任性而为。用好地方立法权，加快推动生态建设和饮用水源保护立法。深化综合行政执法体制机制改革，促进执法力量下沉，加强行政执法监督。落实产权保护制度，维护各种所有制组织和自然人的财产权益。推进行政复议、行政调解规范化建设，依法依规化解行政争议。及时向市委常委会汇报政府党组工作，自觉接受人大及其常委会法律监督和政协民主监督，认真聆听民主党派、工商联、无党派人士和人民团体等社会各界的意见建议，让行政权力在阳光下健康运行。

（三）坚持实干兴政。倡树多为老百姓办实事、做好事的施政理念，双手握紧犁把子、两脚插在墒沟里，在枣庄这片希望的田野上辛勤耕耘，走好转型发展的长征路。政府工作就是抓落实，必须盯紧不落实的事、倒查不落实的人，把“落实、落实、再落实”铭刻在每个政府工作人员的心中。对事关打基础利长远的重大决策、重点工程、重要事项，建立用结果倒逼过程、用过程保障结果的推进机制，引导各级干部脚踏实地勇担当、打起精神扎实干，坚决防止光说不练的“假把式”，切实杜绝蜻蜓点水的“片儿汤”。坚持工作项目化、项目目标化、目标责任化，推动政府部门履职评估考核，力促督查落实常态长效。紧扣想干事、敢干事，建立健全正向激励和容错纠错机制，为实干者撑腰点赞，让干成者受褒奖、得实惠。

（四）坚持从严治政。始终把纪律和规矩挺在前面，严格落实全面从严治党主体责任，认真执行党内政治生活若干准则和党内监督条例，运用好监督执纪“四种形态”。持之以恒落实中央八项规定精神，驰而不息纠正“四风”。牢固树立“过紧日子”的思想，确保“三公”经费只减不增、业务类项目支出整体再压减10%。坚持开正门、堵旁门、关后门，保障公务人员合法合规合理的福利待遇。强化行政监察和审计监督，健全国有资产、公共资金、公共资源监管机制，规范政府采购、工程招投标和政府投资行为。各级政府工作人员必须带头恪守廉洁自律准则，知敬畏、存戒惧、守底线，干净干事、清爽做人，努力营造廉洁高效、务实为民、友善和谐的政务环境。

各位代表！千里之行始于足下，百年梦想成于实干。让我们更加紧密地团结在以习近平同志为核心的党中央周围，在省委、省政府和市委的正确领导下，大力弘扬新时期长征精神，同怀愚公志，共尽精卫责，不忘初心，逐梦前行，为建设自然生态宜居宜业新枣庄而不懈奋斗！
